# Peripheral blood complete remission after splenic irradiation in Mantle-Cell Lymphoma with 11q22-23 deletion and ATM inactivation

**DOI:** 10.1186/1748-717X-1-35

**Published:** 2006-09-06

**Authors:** Andrea Riccardo Filippi, Pierfrancesco Franco, Marco Galliano, Umberto Ricardi

**Affiliations:** 1 Radiation Oncology, University of Torino, Ospedale S. Giovanni Battista, Torino, Italy; 2 Medical Oncology, Ospedale Alba-Bra-ASL 18, Alba-Bra, Italy

## Abstract

Mantle Cell Lymphoma (MCL) is a well-known histological and clinical subtype of B-cell non-Hodgkin's Lymphomas. It is usually characterized by an aggressive disease course, presenting with advanced stage disease at diagnosis and with low response rates to therapy. However few cases of indolent course MCL have been described. We herein report a case of MCL with splenomegaly and peripheral blood involvement as main clinical features. The patient underwent moderate dose splenic radiation therapy and achieved spleen downsizing and peripheral blood complete remission. Splenic irradiation has been extensively used in the past as palliative treatment in several lymphoproliferative disorders and a systemic effect and sometimes peripheral blood complete remissions have been observed. Mainly advocated mechanisms responsible for this phenomenon are considered direct radiation-induced apoptotic cell death, immune modulation via proportional changes of lymphocyte subsets due to known differences in intrinsic radiosensitivity and a radiation-induced cytokine release. The peculiar intrinsic radiosensitivity pattern of lymphoid cells could probably be explained by well-defined individual genetic and molecular features. In this context, among NHLs, MCL subtype has the highest rate of ATM (Ataxia Teleangiectasia Mutated) inactivation. While the ATM gene is thought to play a key-role in detecting radiation-induced DNA damage (expecially Double Strand Breaks), recent in vitro data support the hypothesis that ATM loss may actually contribute to the radiosensitivity of MCL cells. ATM status was retrospectively investigated in our patient, with the tool of Fluorescence In Situ Hybridization, showing a complete inactivation of a single ATM allele secondary to the deletion of chromosomal region 11q22-23. The presence of this kind of cytogenetic aberration may be regarded in the future as a potential predictive marker of radiation response.

## Full text

Mantle-Cell Lymphoma (MCL) has been clearly recognized as a distinct histological and clinical subtype of B-cell non-Hodgkin's Lymphomas. Typical of the elderly, it has an estimated incidence of 2–3/100,000/year and accounts for 8% of all NHLs [1]. Diagnostic work-up usually demonstrates advanced stage disease, often associated with spleen enlargement, bone marrow and peripheral blood involvement [2]. Important clinical prognostic factors are poor PS, splenomegaly, anemia and age [3]. While MCL is generally considered an aggressive disease, with median survivals of 2–3 years, few cases with a fairly indolent disease course are described in the medical literature [4]. We herein report the case of a 90 years-old female referred to our institution hospital, with a history of active phase chronic C-Hepatitis and a 4-yrs established diagnosis of MCL, made upon bone marrow biopsy. The specimen examination demonstrated a nodular pattern of cleaved and small to medium sized cells without residual germinal centres and with loosely structured meshwork of follicular dendritic cells. Immunohistochemistry findings on bone marrow at diagnosis were as follows: CCND1 +, CD 5 +, CD 19 +, CD 20 +, CD 22 +, CD 3 - CD 10 -, CD 23 -, HLA DR +, Surface Membrane IgM-D/K. Flow Cytometry revealed a dual stained population CD5+/CD19 +, CD20+/CD 23 -, CD19 +/CD10 -, FMC7 +. Taking into account those data, expecially CCND1 positivity, we reasonably thought to deal with MCL, instead of other B-cell indolent lymphoproliferative disorders such as Splenic Marginal Zone Lymphoma/Splenic Lymphoma with Villous Lymphocytes. Clinically, her disease course was characterized by a modest splenomegaly, with peripheral blood involvement with marked leucocytosis and lack of lymph node enlargement, and therefore she was repeatedly treated with single-agent chemotherapy (Chlorambucil) with spleen downsizing and normalization of WBC values. At the time of our observation she complained of abdominal pain, anorexia and progressive weight loss. ECOG PS was 1 to 2. Total Body CT scans revealed a massive splenomegaly (25 cm in diameter) without lymph nodes more than 1 cm in diameter at any site. A modest hepatomegaly was also present. CBC resulted as follows: WBC 30.000/mm^3 ^(75 % lymph; 20 % ANC); Hb 12,3 g/dl ; HCT 39 %; PLTs 60.000/mm^3^.

In order to obtain symptomatic relief, we considered radiation therapy and chose to treat the whole spleen to a total dose of 15 Gy in 10 fractions during 2 weeks time, with 6 MV Photons and anterior-posterior parallel opposite fields. Radiation treatment was very well tolerated, without recordable acute toxicity. At clinical and radiological evaluation 3 weeks after RT, complete pain relief was achieved, with reduction in spleen diameter (18 cm at U.S. examination) and, surprisingly (even if already reported), a peripheral blood complete remission. CBC showed: WBC 2100/mm^3^; (ANC 67 %; 19 % Lymph); Hb 12,1 g/dl; HCT 37 %; PLT 61.000/mm^3^. Circulating malignant lymphoid cells were absent at peripheral blood smears and at Flow Cytometry examination. A second bone marrow biopsy was not performed due to patient's age and PS. The latest follow-up performed one year after radiotherapy showed a continuous unmaintained complete peripheral response.

Splenic Irradiation (SI) has been extensively used in the past as palliative treatment in several haematological malignancies such as chronic myeloproliferative disorders [chronic myelogenous leukaemia (CML), essential thrombocythemia (ET), polycythemia vera (PV) and agnostic myeloid metaplasia (AMM)], chronic lymphoproliferative disorders [chronic lymphocytic leukaemia (CLL), prolymphocytic leukaemia (PLL), hairy cell leukaemia (HCL) and splenic marginal zone lymphoma (SMZL)] and even acute myelogenous leukemia [5-11]. Previously reported high response rates could be explained by different mechanisms of action, but the main event is thought to be a direct radiation-induced apoptotic cell death that leads to the elimination of malignant cells located in the spleen (since lymphocytes undergo radiation-induced apoptosis even at very low doses)[12]. A systemic effect and sometimes peripheral blood and even bone marrow complete remissions (CRs) have been observed in several clinical situations [5,13-15], most frequently CLL, PLL and HCL. To our knowledge, no Crs are described during myeloproliferative disorders. Different doses and fraction sizes have been delivered (daily, weekly, three times a week schedules with doses ranging mostly from 5 Gy to 15 Gy). Anyway, the arising question appears to be how SI could clear the bone marrow, removing MCL clones. Several biological mechanisms have been hypothesized by different authors to explain this effect [5]. At first, a direct radiation-induced killing of splenic neoplastic cells has been mentioned, acting through the clearance of a potential source of circulating lymphoma cells [16]. Secondly, an immune modulation via proportional changes of lymphocyte subsets has been advocated as a key event: in this case the differential cell killing of normal lymphocytes (due to known differences in intrinsic radiosensitivity) is believed to cause a redistribution of circulating lymphoid subpopulations with subsequent reduction of normal T-suppressor lymphocytes and increased anti-tumour activity [17,18]. Thirdly, a radiation-induced release of cytokines, such as TNFα or IL-2, is believed to potentially stimulate a secondary immune modulation, enhancing anti-neoplastic cell-mediated effects [19]. In this context, another radiation-induced cell killing mechanism to be considered is the so-called "bystander effect", well described in several experimental studies and anecdotal clinical findings: this phenomenon consists of a biological response of unirradiated neighbours or distant cells after target cells irradiation. When considering distant effects produced by local radiation therapy, it is also known as 'abscopal effect' [20,21]. This event seems to be particularly significant after radiation exposure at low doses and has been advocated to play some kind of role in radiation-induced cancer, radiation damage to healthy tissues and radiation-induced bystander tumor cells killing [22].

The peculiar intrinsic radiosensitivity pattern of lymphoid cells and the above briefly mentioned mechanisms could probably explain the known radiation response phenotype of many lymphoproliferative disorders, but more individual genetic and molecular features could certainly offer more details about some unusual responses of specific patients. Among NHLs, MCL subtype has the highest rate of ATM (Ataxia Teleangiectasia Mutated) inactivation, due to the presence of deletions or mutations in up to 40–50% of patients [23-25]. The ATM gene is thought to play a key-role in detecting radiation-induced DNA damage (expecially Double Strand Breaks) and it is known to be affected by germline mutations (truncation) in patients with Ataxia Teleangiectasia, an autosomal recessive disease characterized by cerebellar ataxia, immunodeficiency, predisposition to lymphoproliferative malignancies and a highly increased sensitivity to ionizing radiations. MCL patients bearing ATM inactivation seem not to have a worse prognosis, while recent in vitro data suggest that ATM loss may actually contribute to radiosensitivity of MCL cells [26]. ATM status was retrospectively investigated in our patient, with the tool of Fluorescence In Situ Hybridization (FISH) on bone marrow biopsy at diagnosis, showing a complete inactivation of a single ATM allele secondary to a deletion of chromosomal region 11q22-23. We suggest that the presence of this kind of cytogenetic aberration, recently reported [27], could be considered as one of the possible explanations of high radiosensitivity profiles of some MCLs, and be regarded in the future as a potential predictive marker of response.

## References

[B1] Campo E, Raffeld M, Jaffe ES (1999). Mantle-Cell Lymphoma. Semin Hemat.

[B2] Witzig TE (2005). Current treatment approaches for Mantle-Cell Lymphoma. J Clin Oncol.

[B3] Andersen NS, Jensen MK, de Nully Brown P, Geisler CH (2002). A Danish population-based analysis of 105 mantle cell lymphoma patients: incidences, clinical features, response, survival and prognostic factors. Eur J Cancer.

[B4] Peghini PE, Fehr J (2002). Analysis of Cyclin D1 expression by Quantitative Real-Time Reverse Transcription-Polymerase Chain Reaction in the diagnosis of Mantle Cell Lymphoma. Am J Clin Pathol.

[B5] Weinmann M, Becker G, Einsele H, Bamberg M (2001). Clinical indications and biological mechanisms of splenic irradiation in chronic leukaemias and myeloproliferative disorders. Radiother Oncol.

[B6] Wagner H, McKeough PG, Desforges J, Madoc-Jones H (1986). Splenic irradiation in the treatment of patients with chronic myelogenous leukemia or myelofibrosis with myeloid metaplasia. Results of daily and intermittent fractionation with and without concomitant hydroxyurea. Cancer.

[B7] De Rossi G, Biagnini C, Lopez M, Tombolini V, Mandelli F (1982). Treatment by splenic irradiation in 22 chronic lymphocytic leukemia patients. Tumori.

[B8] Elliott MA, Tefferi A (1999). Splenic irradiation in myelofibrosis with myeloid metaplasia: a review. Blood Rev.

[B9] McFarland JT, Kuzma C, Millard FE, Johnstone PA (2003). Palliative irradiation of the spleen. Am J Clin Oncol.

[B10] Muncunill J, Villa S, Domingo A, Domenech P, Arnaiz MD, Callis M (1990). Splenic irradiation as primary therapy for prolymphocytic leukemia. Br J Hematol.

[B11] Sharp RA, MacWalter RS (1983). A role for splenic irradiation in the treatment of hairy-cell leukaemia. Case report and review of the literature. Acta Haematol.

[B12] Thomson AE, Vaughan-Smith S, Peel WE, Wetherley-Mein G (1985). The intrinsic radiosensitivity of lymphocytes in chronic lymphocytic leukaemia, quantitatively determined independently of cell death rate factors. Int J Radiat Biol Relat Stud Phys Chem Med.

[B13] Sgarabotto D, Vianello F, Radossi P, Poletti A, Sotti G, Stefani PM, Sartori R, Girolami A (1997). Remission in hairy-cell leukaemia-variant following splenic radiotherapy alone. Leuk Lymphoma.

[B14] Chisesi T, Capnist G, Dal Fior S (1991). Splenic irradiation in chronic lymphocytic leukaemia. Eur J Hematol.

[B15] Kiss A, Haubenstock A, Bognar H, Scheiderbauer R, al-Mobarak M, Base W (1989). Splenic irradiation as primary therapy for prolymphocytic leukaemia. Am J Hematol.

[B16] Delic J, Magdelenat H, Barbaroux C, Chaillet MP, Dubray B, Gluckman E, Fourquet A, Girinsky T, Cosset JM (1995). In-vivo induction of apoptosis in human lymphocytes by therapeutic fractionated total body irradiation. Br J Radiol.

[B17] Paule B, Cosset JM, Le Bourgeois JP (1985). The possible role of splenic irradiation in chronic lymphocytic leukaemia: a critical review. Radiother Oncol.

[B18] De Ruysscher D, Waer M, Vandeputte M, Aerts R, Vantongelen K, van der Schueren E (1992). Changes of lymphocyte subsets after local irradiation for early stage breast cancer and seminoma testis : Long term increase of activated (HLA-DR +) T cells and decrease of 'naive' (CD4/CD45R) T lymphocytes. Eur J Cancer.

[B19] Bessler H, Notti I, Cohen AM, Klein B, Djaldetti M (1994). Inhibition of leukemic cell proliferation by factor (s) released from irradiated lymphocytes of B-chronic lymphocytic leukaemia patients. Am J Hematol.

[B20] Little JB (2006). Cellular radiation effects and the bystander response. Mut Res.

[B21] Kaminski JM, Shinohara E, Summers JB, Niermann KJ, Morimoto A, Brousal J (2005). The controversial abscopal effect. Cancer Treat Rev.

[B22] Prise KM, Schettino G, Folkard M, Held KD (2005). New insights on cell death from radiation exposure. Lancet Oncol.

[B23] Schaffner C, Idler I, Stilgenbauer S, Dohner H, Lichter P (2000). Mantle cell lymphoma is characterized by inactivation of the ATM gene. Proc Nat Acad Sci USA.

[B24] Camacho E, Hernandez L, Hernandez S, Tort F, Bellosillo B, Bea S, Bosch F, Montserrat E, Cardesa A, Fernandez PL, Campo E (2002). ATM gene inactivation in mantle cell lymphoma mainly occurs by truncating mutations and missense mutations involving the phosphatidylinositol-3 kinase domain and is associated with increasing numbers of chromosomal imbalances. Blood.

[B25] Fang NY, Greiner TC, Weisenburger DD, Chan WC, Vose JM, Smith LM, Armitage JO, Mayer RA, Pike BL, Collins FS, Hacia JG (2003). Oligonucleotide microarrays demonstrate the highest frequency of ATM mutations in the mantle cell subtype of lymphoma. Proc Nat Acad Sci USA.

[B26] M'kacher R, Bennaceur A, Farace F, Lauge A, Plassa LF, Wittmer E, Dossou J, Violot D, Deutsch E, Bourhis J, Stoppa-Lyonnet D, Ribrag V, Carde P, Parmentier C, Berheim A, Turhan AG (2003). Multiple molecular mechanisms contribute to radiation sensitivity in mantle cell lymphoma. Oncogene.

[B27] Stilgenbauer S, Winkler D, Ott G, Schaffner C, Leupolt E, Bentz M, Moller P, Muller-Hermelink HK, James MR, Lichter P, Dohner H (1999). Molecular characterization of 11q deletions points to a pathogenetic role of ATM gene in mantle cell lymphoma. Blood.

